# Bicolumnar 90-90 Locking Plate Fixation Through a Posterior Approach for the Treatment of Low Distal Humeral Fracture in Elderly Patients

**DOI:** 10.7759/cureus.40123

**Published:** 2023-06-08

**Authors:** Abdulrahim Dündar

**Affiliations:** 1 Department of Orthopedics and Traumatology, Hitit University Erol Olçok Training and Research Hospital, Çorum, TUR

**Keywords:** posterior approach, elderly patients, double plating, orthogonal, humeral fractures, low transcondylar

## Abstract

Background and objective

Treating transcondylar humeral fractures in elderly individuals remains a challenge in trauma surgery, with plate fixation being one potential treatment method. This retrospective study was conducted to assess the effectiveness of plate fixing through a posterior approach in elderly individuals suffering from distal humeral fractures.

Methods

This retrospective study involved 28 older participants aged ≥65 years with low transcondylar fractures of the humerus (AO/OTA 13A2-3 fractures). We used the 90-90 orthogonal method for treatment. The inclusion criteria were as follows: (1) low transcondylar type of distal humeral fractures (13A2-3 according to the AO/OTA classification system), (2) patients aged ≥65 years, and (3) a follow-up period of at least 12 months. The exclusion criteria were as follows: polytrauma, pathological injuries, chronic elbow osteoarthritis or degenerative arthropathy, and fractures impacting the articular surface of the distal humeral. Clinical outcomes were assessed in terms of the visual analog scale (VAS) score, Mayo Elbow Performance Score (MEPS), and range of motion (ROM) of the elbow joint.

Results

The mean age of the patients was 72.25 years (range: 65-81 years), of which 14 (50%) were female and 14 (50%) were male. The mean VAS score for pain was 2.7 (range: 0-6). The mean angle of flexion was 130.6^o^ (range: 115-140^o^), and the mean angle of the extension was −27.7 (range: −21 to −34). Regarding MEPS, 23 patients had an excellent score, four patients had a good score, and one patient had a poor score. There were four complications (two major and two minor) in the patients involved in the study.

Conclusion

Based on our findings, 90-90 plate fixation for low distal humeral fractures is associated with a high union rate and satisfactory clinical outcomes. Although we found complications in four patients, their healing was not affected. Therefore, we concluded that through improved monitoring and care, we could overcome such complications, and they did not affect the healing of the bone.

## Introduction

Although low distal humerus fractures are uncommon and account for less than 2% of all fractures, they are more common in older people. The peri-articular position, the tiny diameter of distal bone fragments, and the osteoporotic nature of the bone in older persons make fractures of the distal third of the humerus problematic injuries. The intricacy of such fractures and the low bone density in older individuals lead to difficulties in internal fixation. Therefore, surgical and implantation choices require extra care and consideration. Many researchers now recommend first-line total elbow arthroplasty (TEA) in properly chosen patients due to the technical challenges caused by these fractures and the risk of unstable fixation [1]. To reduce the risk of non-union and enable early elbow mobility, which is essential for successful treatment outcomes, internal fixation in older individuals must be exceedingly secure. The biomechanical evaluation of different fixation constructions has received fresh attention as a result of the debate over fixation methods and the launch of newly designed implants such as pre-contoured plates and locking plates [2]. Systems using locking plates have proven successful in stabilizing constructions in brittle bone and preserving fracture reductions. Thus, these methods appear to be suitable for treating older individuals [3].

Currently, the most common operative method for managing these fractures is open reductions with double plate fixation [4]. Double plating, which involves using plates on both the lateral and medial columns and positioning them either perpendicularly or parallel to one another, is the conventional fixation approach. Generally, there are two methods for the fixation of the low transcondylar fracture pattern: the parallel plating method and the 90-90 (orthogonal) plating method. We use the 90-90 orthogonal method. Some surgeons use medial and lateral double incisions for fixation, while others use only one incision with a posterior approach. There is a scarcity of data regarding the results of open reduction and internal fixation (ORIF) with an anatomical plate for the treatment of low transcondylar fractures of the distal humerus. In light of this, the aim of this study was to present the results of the bicolumnar 90-90 plating construct technique using only the single-incision approach for ORIF of distal humerus fractures.

## Materials and methods

This retrospective study was conducted at the Orthopedic and Traumatology Department of a level 3 tertiary training and research hospital in Turkey between 2016 and 2021, and approved by the local institutional review board (approval no: 2022-28). Informed consent was obtained from all the patients who participated in the study: 28 older participants aged >65 years with low transcondylar humerus fractures (13A2-3 fractures according to the AO/OTA classification). We aimed to evaluate the radiological and clinical results and complications of double-plate fixation through a single incision with a posterior approach. The inclusion criteria were as follows: patients aged 65 years of age or older, with transcondylar humeral fractures classified as 13A2-3 fracture patterns by the AO/OTA, and with a follow-up for at least 12 months following surgery. The exclusion criteria were as follows: polytrauma, pathological injuries, chronic elbow osteoarthritis or degenerative arthropathy, and fractures impacting the articular surface of the distal humerus.

**Figure 1:**
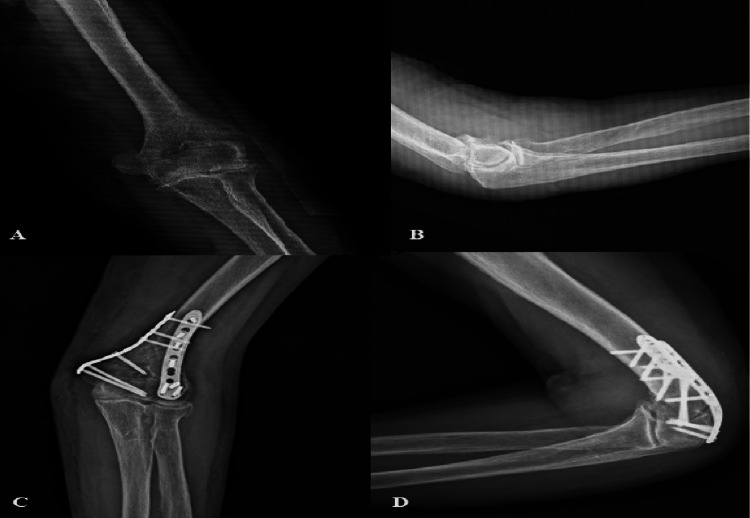
78-year-old male with a displaced low transcondylar fracture (AO/OTA 13A2-3) (A) Preoperative anterioposterior and (B) lateral radiographs of a 78-year-old male patient with a displaced low transcondylar fracture. (C, D) Postoperative anteroposterior and lateral radiographs showed a stable fixation with an orthogonal pattern before the completion of follow-up

**Figure 2:**
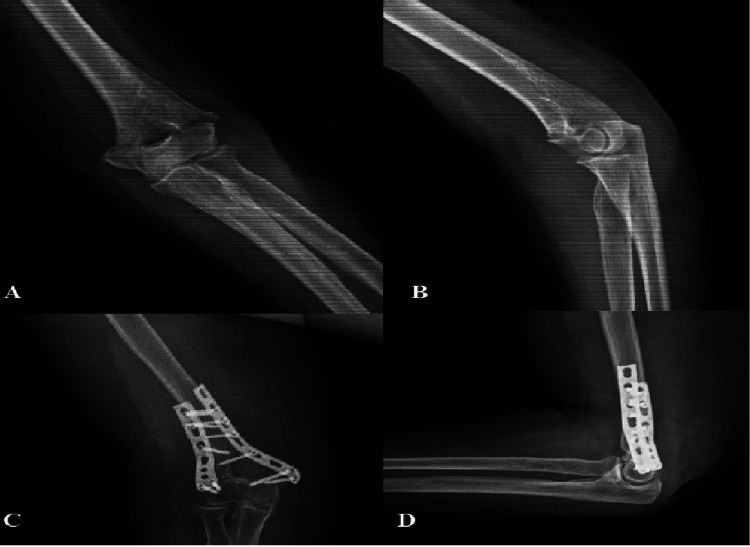
80-year-old female with a low transcondylar fracture (A) Preoperative anteroposterior and (B) lateral radiographs of a low transcondylar fracture (80-year-old female). (C, D) Postoperative anteroposterior and lateral images at four months; the union was achieved successfully

All procedures were performed with the patient in the lateral decubitus position under brachial plexus anesthesia and general anesthesia depending on their general condition. A curved posterior incision was made in the distal elbow. The ulnar nerve was identified but was routinely transposed. After reducing the fragments of the fracture, Kirschner wires were used for temporary fixation. Double plates were placed as both posterolateral and medial columns in an orthogonal position (Figures 1-2). A long-arm cast or splints were applied for two weeks. After suture removal, active physiotherapeutic mobilization without load was initiated at two weeks. During the follow-up period, standard anteroposterior and lateral radiographs were obtained. All patients were evaluated one year after the surgery at the least and assessed in terms of the Mayo Elbow Performance Score (MEPS), visual analog scale (VAS) score, and range of motion (ROM) activity. MEPS was used to evaluate the elbow function at the end of the follow-up, with the results categorized as excellent (>90), good (75-89), fair (60-74), and poor (<60). Instances of complications, loss of reduction, screw loosening, ulnar nerve disturbance, and infection were recorded.

## Results

The average age of the patients was 72.25 years (range: 65-81 years), of which 14 (50%) were female and 14 (50%) were male. In 15 patients, the right humerus was affected, while the left humerus was affected in 13 patients. In 16 patients, the mechanism of injury was a fall down the stairs, and it was a fall from a ladder in 12 patients, which were mostly low-energy injuries. According to AO/OTA classification, all patients were with low transcondylar (AO Type 13A2-3) fractures of the humerus. All patients were treated with a single posterior approach, and the orthogonal fixation method was used. The average operation time was 72 minutes (range: 54-110 minutes), and the mean follow-up time was 15 weeks (range: 12-20 weeks), as seen in Table 1.

**Table 1:** Demographic and surgical data

Variable	Values
Number of patients	28
Age, years, mean (range)	72.25 (65-81)
Fracture pattern, n (%)	
AO/OTA 13A2-3	28 (100)
Side, n	
Right	15
Left	13
Injury mechanism, n (%)	
Fall	12 (42.8)
Slip-down	16 (57.1)
Fixation method: orthogonal, n (%)	28 (100)
Operation time, minutes, mean (range)	72 (54-110)
Sex, n (%)	
Male	14 (50)
Female	14 (50)
Follow-up time, week, mean (range)	15 (12-20)

Radiological and clinical results 

The average union time was 72.07 days (range: 54-110 days). The average VAS score was 2.7 (range: 0-6). The mean angle of flexion was 130.6 (range: 115-140), and the average angle of the extension was −27.7 (range: −21 to −34). Regarding MEPS, 23 patients had an excellent score, four patients had a good score, and one patient had a poor score.

Complications

There were four complications (14.2%) in the patients involved in this study: two major complications and two minor complications. Regarding minor complications, after four months following surgery, a significant consequence in one case was the degeneration of the skin above the ulnar plate. The material was removed and the skin was rebuilt when the injury had entirely healed. The other patient with a minor complication had delayed union, as indicated by fracture lines. As for major complications, ulnar nerve disturbance was present in two patients diagnosed with ulnar neuropathy. In one patient, a second surgery was considered due to the ulnar nerve disturbance, but they refused the revisional surgery due to a poor general condition, as seen in Table 2.

**Table 2:** Clinical and radiological outcomes in patients with distal humeral fractures SD: standard deviation; VAS: visual analog scale

Radiologic parameter	Values
Time to the radiographic union, days, mean ± SD	72.07 ± 10
VAS score, mean ± SD	2.7 ± 1.5
Flexion range, mean ± SD	130 ± 5.8
Extension range, mean ± SD	-27.7 ± 4.3
Mayo score, n	
Poor	1
Good	4
Excellent	23
Ulnar nerve transposition, n (%)	28 (100)
Complication, n (%)	4 (14.2)
Skin necrosis	1 (3.6)
Delayed union	1 (3.6)
Ulnar nerve disturbance	2 (7.1)

## Discussion

Low distal humeral fractures occur primarily in the geriatric osteoporotic population [5]. As non-operative treatment has been found to carry a significant risk of functional loss, the surgical method is widely considered to be the gold standard for treating juxta-articular and intra-articular distal humeral injuries. However, even if such operations are functionally effective, some lingering impairments (e.g., tightness and weakening in the elbow joint) may still exist [6]. Frankle et al. [7] compared two groups of geriatric patients that underwent ORIF and TEA, where the fixation failure rate of ORIF was 25% and the complication rate of TEA was 25%, which suggested that the best method for low distal humeral fracture must be personalized based on each individual's fracture pattern. Aldridge et al. [8] showed that TEA significantly reduced pain and restored adequate function in elderly patients. However, some complications of TEA have been reported, such as prosthetic loosening, periprosthetic elbow fracture, and superficial and deep infection. Therefore, TEA may be an excellent salvage technique for low distal fractures that are unsuitable for internal fixation.

The posterior single-incision approach was employed previously by Daniel et al. [9], who, in their investigation, used a new technique for improving distal stabilization in these injuries in 15 individuals with bicolumnar 90-90 plating constructs. With an overall range of movement of 105, 14 participants had radiological fusion after approximately 77 days following surgery. These results are similar to those found in our research. Simone et al. [10] treated patients with low transcondylar fracture by ORIF and found a delayed union rate of 29%. However, in our study, we found only one case of delayed union (3.1%), and most fractures healed completely.

It is now generally recognized that optimal results can be achieved by ORIF with a posterior approach [11]. In the current study, 90-90 orthogonal plating appeared to be the ideal option for early postoperative rehabilitation of the elbow in geriatric patients. This finding is comparable with that of a previous study [12]. Several previous biomechanical reports have shown that the double plate fixation method provides adequate and rigid stabilization in most fracture patterns and is, therefore, recommended for low distal humeral fractures [13,14]. However, ulnar nerve disturbance associated with double plate osteosynthesis has been frequently reported [15]. In our study, ulnar nerve disturbance was found in two patients diagnosed with ulnar neuropathy.

We believe that the 90-90 orthogonal method may be the best choice for the treatment of low distal humeral fracture in elderly patients. Our study findings also correlated with those of Korner et al. [6], who conducted a retrospective clinical analysis of 45 participants with surgically treated distal humerus injuries (median age: 73 years). Although they reported a high complication rate, including screw loosening and implant failure at the side of the lateral column, they also observed good or excellent functional results.

This study has several limitations. Firstly, it employed a retrospective design. Secondly, it had a smaller patient population. We recommend further prospective randomized studies comparing orthogonal, parallel, and TEA methods to establish the best course of action for low distal humeral fractures.

## Conclusions

Our findings showed that internal fixing in older individuals was appropriate regardless of deteriorated bone density and a significant risk of complications. We determined that for humeral fractures, 90-90 orthogonal plate fixation is best performed by using a single-incision posterior approach. Most elderly patients with low distal humeral fractures can be successfully treated with 90-90 orthogonal ORIF, without the need for TEA.
